# Contextual Variation in External and Internal Workloads across the Competitive Season of a Collegiate Women’s Soccer Team

**DOI:** 10.3390/sports9120165

**Published:** 2021-12-08

**Authors:** Lauren E. Rentz, William Guy Hornsby, Wesley J. Gawel, Bobby G. Rawls, Jad Ramadan, Scott M. Galster

**Affiliations:** 1 Human Performance Innovation Center, Rockefeller Neuroscience Institute, West Virginia University, Morgantown, WV 26505, USA; Brawls@hsc.wvu.edu (B.G.R.); Jramadan@hsc.wvu.edu (J.R.); Scott.Galster@hsc.wvu.edu (S.M.G.); 2 College of Physical Activity and Sport Sciences, West Virginia University, Morgantown, WV 26505, USA; William.Hornsby@mail.wvu.edu (W.G.H.); wjg00003@mix.wvu.edu (W.J.G.)

**Keywords:** GPS, workload, load monitoring, athlete, collegiate sport, soccer

## Abstract

As sports technology has continued to develop, monitoring athlete workloads, performance, and recovery has demonstrated boundless benefits for athlete and team success. Specifically, technologies such as global positioning systems (GPS) and heart rate (HR) monitors have granted the opportunity to delve deeper into performance contributors, and how variations may exist based upon context. A team of NCAA Division I women’s soccer athletes were monitored during games throughout one competitive season. Individual athlete, positional groups, and team external and internal workloads were explored for differences based upon game location, opponent ranking, game result, and the final score differential. Game location and opponent ranking were found to have no effect on team-wide absolute or relative external workloads, whereas game result and score differential did. Internal workloads across the team tended to only vary by game half, independent of game context; however, the HR of defenders was determined to be higher during losses as compared to wins (*p* = 0.0256). Notably, the games that resulted in losses also represented the games with the fewest number of substitutions. These findings suggest high value in monitoring performance and workloads that are characteristic of varying, often multifaceted, contexts. It is hoped that this information can lead to more informed approaches to vital game-time and coaching decisions.

## 1. Introduction

Over the last two decades, the use of integrated, practitioner-driven sport science applications have grown exponentially. This rise has occurred across many sporting endeavors (individual sport, team sport) and across many levels of sport (international Olympic sport, collegiate, professional). Specifically, within the global football community (or soccer as it is referred to in the United States and thus will be referred to herein), athlete monitoring systems aimed at providing coaches and sport scientists a better “view” of their athletes’ current physiological state has become commonplace. This better understanding of the work athletes have performed is especially sought out within the professional and higher-level club (international) or collegiate (USA) soccer domains.

Historically, pioneers of load monitoring such as Borg [1,2], Banister [3], Edwards [4] and Foster [5] studied and utilized various strategies collecting subjective load scores (e.g., session rating of perceived exertion [RPE]) and/or objective load scores (e.g., heart rate, lactate) in an attempt to better understand the amount of work performed along with the athlete’s internal physiological response. Indeed, strategies such as session RPE, a low-cost option that continues to be very useful for coaches and sport scientists interested in load monitoring [6,7]. With the advent of wearable technology in sport, load monitoring often now involves wearable instruments such as accelerometers and gyroscopes along with more user-friendly heart rate (HR) monitors.

A current staple of an athlete monitoring system for the sport of soccer is the use of global positioning systems (GPS) and HR monitors to provide both an external and internal load assessment during soccer related activities, such as during games, practices, and training. Concomitantly, sport science research devoted to this area of athlete monitoring has sharply risen over the last several years. Specifically, for research devoted to athlete locomotion assessment during exercise, Malone et al. [8] notes that from 2001 to 2018 research has escalated from 3 to 136 published articles per year. While this general area of research is now quite robust, when dissecting through specific attributes of the existing research to better understand variations based on sex and level of competition within a sport, the amount of relevant literature can drastically descend. For example, existing research is exponentially reduced when considering only motion analysis research, all of which widely spans across leagues at the elite and sub-elite level [9,10,11,12,13,14,15,16,17,18,19,20,21,22]. Further reductions occur when identifying research conducted on U.S. collegiate Division I female soccer players [9,10,21,22].

Investigations that capture multiple games or an entire season provide a more robust picture of physiological demands and allows for greater contextualization. While there are many season-long studies on male soccer players [23,24,25], there are far fewer published investigations within Division I women’s college soccer. Junior et al. [9] and Wells et al. [21] each reported on the monitoring of a single season, while Sausaman et al. [10] impressively covered 4 consecutive seasons from a collegiate women’s team. Although each of these are critically important studies for the field, these three did not report heart rate and the athletes were from non-“Power 5” athletic programs.

A vital perspective to consider for athlete monitoring is that an individuals’ idiosyncratic response to a given external load is likely to vary based upon the context of the situation. More specifically, both the mental and physiological demands of a game can largely impact the execution of a task, with the level of difficulty tending to increase as conditions deviate further away from optimal training conditions [22,26,27]. The quantification of internal load is often achieved via subjective reports, which can be more greatly skewed toward perceived demands, or objectively via HR or heart rate variability (HRV) [26,28]. For this reason, internal workload can be highly valuable for monitoring the relationship between external demands, typically set forth by the game, and athlete demands; garnering a more comprehensive understanding of individual stress may demonstrate greater utility for monitoring fatigue across various game scenarios [26,27,28,29].

Thus, the focus of the present study is to present observational data following a female collegiate soccer team across an entire collegiate season. Given the observational and descriptive nature of the study, this research was deemed hypothesis generating. Generally, the authors were interested in attaining a better understanding of external and internal workloads throughout a season along with potential differences and trends between different position groups, lineup configurations (e.g., substitutions), and various influential tactical elements. Based on previous studies, the authors of the present study anticipated that various factors related to increased work performed would lead to increased internal stress.

Specifically, exploratory trends were compared between broad situational factors, most of which have been previously evaluated, including game location, game result, rigor of the opponent, and final score differential [30,31,32,33,34]. Additional noteworthy aspects include the inclusion of average heart rate, which has only previously been considered by a few studies [11,12], as well as the high quality of the both the team captured and their opponents. To emphasize the success and high level of play, the team evaluated herein is a member of a National Collegiate Athletic Association (NCAA) “Power 5” conference, maintained a top ten ranking throughout the season of reference, and has a long history of NCAA national tournament appearances.

## 2. Materials and Methods

### 2.1. Participants

All players of an NCAA Division I collegiate women’s soccer team volunteered to participate in the study, each providing their written informed consent to participate. The study procedures were approved by the West Virginia University Institutional Review Board and followed all ethical principles set forth by the Declaration of Helsinki.

Because coaching decisions were not dictated by the study, and were rather only observations, only 18 of these players participated in field-based play throughout the season; thus, these 18 players, which comprised all contributing players aside from goalkeepers, were included in analysis (age, 19.9 ± 1.1 years; body mass, 63.9 ± 6.0 kg; height, 170.1 ± 5.6 cm; leg length, 88.6 ± 4.6 cm).

### 2.2. Wearable Sensors

Athletes were monitored during games via GPS and local positioning system (LPS), inertial movement data, and HR monitors. External workloads encompassing GPS, LPS, and inertial data were captured using the Vector S7 device made by Catapult Sports (Catapult Sports, Melbourne, Australia), which were positioned on each athlete in the center of their upper back. The Vector S7 includes a tri-axial accelerometer, tri-axial gyroscope, and tri-axial magnetometer, each provided at sampling rates of 100 Hz, whereas GPS and LPS are sampled at 10 Hz. Similar Catapult devices have previously been found to have high rates of reliability [35,36,37]. The device connects wirelessly to a Polar H7 (Polar Electro Inc., Lake Success, NY, USA), which is equipped on the athlete using an elastic chest strap; this device is used to derive electrocardiogram (ECG) parameters for calculations of HR. All data is synced to Catapult’s Openfield software following each session for data analytics.

### 2.3. Data Collection

Each player was assigned a Vector S7 and Polar H7 device, which remained consistent throughout the season. Sensors were worn during each game and were individually timestamped each time a player checked in or out of the game to ensure data was only analyzed during active periods for each player. This data is concurrently collected as a standard practice of the team and is a part of a larger on-going athlete monitoring effort that has been established for several years.

Due to enhanced safety concerns relative to the COVID-19 pandemic, many seasons were condensed and had altered schedules as compared to traditional collegiate soccer seasons. Only conference opponents were played, which comprised nine games, each occurring exactly one week apart (+/− an hour for time zone differences). Data was collected in field-based players (all players other than goalkeepers) and included 18 players in total throughout the nine conference games. Descriptive information, final results, and number of field-based players utilized during each game are provided in Table 1 in order of schedule. As many as 17 field players played in a single game, and as few as 13.

### 2.4. Measures and Variables

#### 2.4.1. Objective Workload Variables

**Distance [m]**—Defined as the total distance, in meters, traveled during active play.

- **Absolute Summated Distance [m]**—Summated distances encompass the combined number of meters traveled by the team or a positional group for a given session (full game or half). It accounts for contributions made by all utilized players of the session as a measure of group external workload.
- **Absolute Average Distance [m]**—Average distances represent the mean number of total meters traveled during a session (full game or half) across the team or positional group. It represents the average contributions per player.
- **Meters per Minute (M/min) [m/min]**—A relative measure of external workload; the number of meters traveled by a player during the session (full game or half) normalized by their active game duration. A relative measure of distances traversed that can be compared between players contributing only a few minutes and those playing the full game.

**Player Load [au]**—Used to quantify external loads, player load (quantified in arbitrary units, au) is a calculation used by Catapult Sports that is calculated by summating instantaneous accelerations across all three planes and scaling by a factor of 100 [38]. While player load has been known to correlate with distances traveled, it additionally accounts for the variations in speed and frequency of movements [39].

- **Absolute Summated Player Loads [au]**—Summated player load encompass the combined number of arbitrary units recorded in the team or a positional group for a given session (full game or half). It accounts for contributions made by all utilized players of the session as a measure of group external workload.
- **Absolute Average Player Loads [au]**—Average player loads represent the mean number of total arbitrary units recorded during a session (full game or half) across the team or positional group. It represents the average contributions per player.
- **Player Load per Minute (PL/min) [au]**—A relative measure of external workload; the number of arbitrary units recorded by a player during the session (full game or half) normalized by their active game duration. A relative measure of player load that can be compared between players contributing only a few minutes and those playing the full game.
- **Player Load per Meter (PL/M) [au/m]**—A relative measure of external workload; the number of arbitrary units recorded by a player during the session (full game or half) normalized by their total distance traveled. A relative measure of player load that can be compared between players contributing smaller vs. greater efforts, which is representative of the magnitude and frequency of velocity changes standardized to a relative distance.

**Heart Rate (HR) [BPM]**—Heart rates recorded using the Polar H7 were averaged throughout each player’s active time for each session (full game or by half) and were used to quantify internal workload. The age of all players were within 3.3 years, and thus, they each had very similar age predicted maximum heart rates.

#### 2.4.2. Contextual Variables

**Game Location**—Games were classified as either *home* or *away*; no games included herein occurred at a neutral site. Four games occurred at home, spanning 59 records, and five games were away, spanning 71 records across all 18 players.

**Opponent Ranking**—Games were classified as either having a *ranked* or *unranked* opponent based upon the Top 25 NCAA Coaches Poll at the time of play. Three games were against ranked opponents, spanning 41 records, and six were against unranked opponents, spanning 89 records.

**Game Result**—Games were classified as either a *win* or a *loss* based upon the final result. Seven games resulted in a win, spanning 104 records, and two games resulted in a loss, spanning 26 records.

**Score Differential**—Games were classified as having a final score differential of either *two or more* or *within one*, regardless of who the winning team was. Two games had a differential of two or more, which were both wins and spanned 32 records, whereas seven games were within one, which spanned 98 records and included five wins and two losses.

### 2.5. Statistical Analysis

Catapult data was exported from the Catapult Openfield cloud and compiled in Microsoft Excel (version 16). Final game statistics were compiled from official NCAA results. Statistical analyses were completed using JMP Pro 14 software (SAS Institute, Cary, NC, USA).

Eight of the nine games were decided at the end of regulation, whereas a tie existed at the end of regulation for the remaining game, causing it to extend into second overtime; for consistency of data comparisons, only the first 90 min of each game were included herein.

Independent t-tests and two-way mixed model Analysis of Variance (ANOVA) analyses were utilized to determine team-based differences in total workloads of the two halves based upon the various contextual criteria. All two-way ANOVA’s were run with three fixed effects: main effect of *half*, main effect of *contextual variable*, and Interaction between half*contextual variable. Aside from assessments of summated absolute workloads, a random effect of *opponent* nested by *player* was applied to mixed model ANOVAs. Team-wide individual player data was checked across all variables for unequal variance using the Bartlett Test and was determined to have equal variance; as such, a residual covariance structure was utilized for constructing model effects. Multiple comparisons were corrected using Tukey’s HSD when identifying significance of pairwise comparisons, and effect size was calculated using Hedges *g*.

Additional two-way mixed model ANOVA’s were utilized to determine whether differences existed during regulation between positional groups based upon the defined contextual criteria. These analyses were again run with three fixed effects: main effect of *position*, main effect of *contextual variable*, interaction between position*contextual variable. Multiple comparisons were corrected using Tukey’s HSD when identifying significance of pairwise comparisons, and effect size was calculated using Hedges *g*.

## 3. Results

### 3.1. Team Analysis

Across the team, all players averaged 64.59 ± 27.0 min throughout the first 90 min of regulation of all games. Average minutes played was significantly lower during games that were won (62.21 ± 27.3 min) versus games that were lost (74.09 ± 23.8 min, t(43.1) = −2.21, *p* = 0.0324, *g* = 0.19).

There was no difference in player duration based upon game location, opponent rank, or score differential.

#### 3.1.1. Absolute Workload

Fixed effects from mixed model ANOVA’s for averages and summated distances and player loads for the full team are included in Table 2. Multiple analyses demonstrated significant main effects of half on absolute workload metrics, all of which demonstrate trends of higher workloads in the first half.

Game location and opponent rank had no effect on absolute workloads.

Game result significantly affected both average distances and player loads, but not summated team workloads, with athletes tending to perform at higher absolute workloads in games that resulted in a loss. Comparatively, a significant main effect existed for score differential on summated player loads, demonstrating higher total team loads when the final score was determined by 2 or more points (F(1,14) = 4.69, *p* = 0.0481).

#### 3.1.2. Relative Workload

Mean relative workloads from the first 90 min of all games are summarized in Table 3.

Full team analyses by mixed model ANOVA’s assessing relative workloads by half are included in Table 4. All analyses evaluating PL/min and M/min demonstrated a significant main effect of half, with higher workloads consistently occurring in the first half (all *p* < 0.0001); however, no significant main effects or interactions existed for any analysis of PL/meter.

Neither game location nor opponent rank had an effect on any measure of relative workload.

In addition to main effects on half, significant interactions between result*half existed for PL/min (F(1,116.2) = 12.32, *p* = 0.0006) and M/min (F(1,115.5) = 13.22, *p* = 0.0004). Pairwise comparisons also demonstrated significantly lower workloads during the second half of wins, as compared to the first, for PL/min (t(116.2) = 8.24, *p* < 0.0001, *g* = 0.72) and M/min (t(115.5) = 9.12, *p* < 0.0001, *g* = 0.80), whereas the two halves did not differ during losses.

Score differential also impacted PL/min and M/min, in addition to half; significant main effects of score differential existed for PL/min (F(1,127.6) = 8.67, *p* = 0.0038) and M/min (F(1,126.7) = 7.13, *p* = 0.0086), with higher workloads consistently recorded from games won by two or more points. Pairwise differences existed in PL/min between the first and second halves of both games within one point (t(117.2) = 6.53, *p* < 0.0001, *g* = 0.57) and games decided by two or more (t(117.2) = 2.89, *p* = 0.0234, *g* = 0.25); additionally, significantly higher PL/min were recorded in games won by two or more points in both the first (t(117.2) = 2.66, *p* = 0.0431, *g* = 0.23) and second (t(117.2) = 3.01, *p* = 0.0165, *g* = 0.26) halves. Similarly, M/min were significantly higher in the first half of both games that were within one point (t(117) = 7.20, *p* < 0.0001, *g* = 0.63) and games won by two or more (t(117) = 3.46, *p* = 0.0041, *g* = 0.30); however, only the second half significantly differed in M/min based upon score differential (t(117) = 2.69, *p* = 0.0408, *g* = 0.24).

#### 3.1.3. Internal Workload

Across all nine games, average heart rate was found to be significantly higher during the first half (171.6 ± 8.9 BPM) than the second half (169.1 ± 8.2 BPM; t(117.1) = 4.42, *p* < 0.0001, *g* = 0.39). No differences in team-wide heart rate were found to exist based upon game location, opponent rank, game result, or final score differential, however all analyses demonstrated significant main effects of half; all fixed effects from mixed model ANOVA’s can be seen in Table 5.

### 3.2. Positional Analysis

During the first 90 min of regulation, defenders averaged 89.2 ± 14.28 min, midfielders 47.0 ± 21.1 min, and forwards 66.3 ± 24.0 min per game; a one-way ANOVA found these durations to be significantly different (F(2,127) = 49.26, *p* < 0.0001) with significant pairwise differences between each positional group (*p* < 0.0001).

#### 3.2.1. Absolute Workload

**Figure 1:**
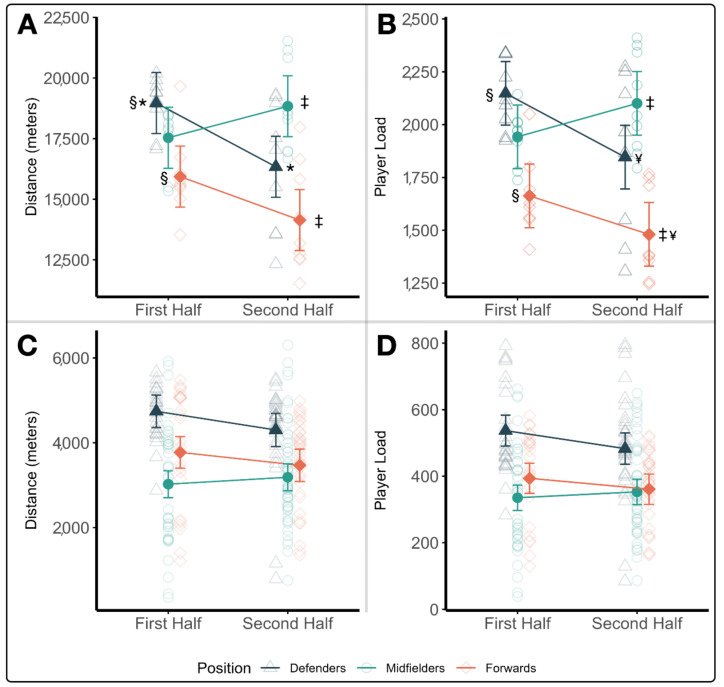
(**A**–**D**). Absolute workload trends throughout the season. Least squares means plots demonstrating positional trends in absolute external workloads across the nine games. (**A**) Summated distance for each game assessed by position group. (**B**) Summated player loads for each game assessed by position group. (**C**) Average distances by all players within a position group. (**D**) Average player loads by all players within a position group. (*) denotes significant pairwise differences between the first and second half workloads in defenders, (§) denotes significant pairwise differences between defenders and forwards during the first half, (‡) denotes significant pairwise differences between midfielders and forwards in the second half, and (¥) denotes significant pairwise differences between defenders and forwards in the second half.

The average sums of distance and player load are provided in Table 6 for each position during the two halves of all nine games. In general, defenders and forwards trended towards having lower absolute workloads during the second half, while midfielders tended to have higher workloads in the second half as compared to the first, as seen in Figure 1A,B. This differed from average distance and player load by players of the various positions, which is displayed in Figure 1C,D. Recorded average workloads tended to be the highest for defenders in both halves as compared to midfielders (distance: t(242) = 7.74, *p* < 0.0001, *g* = 0.68; PL: t(242) = 7.57, *p* < 0.0001, *g* = 0.66) and forwards (distance: t(242) = 4.55, *p* < 0.0001, *g* = 0.40; PL: t(242) = 5.64, *p* < 0.0001, *g* = 0.49). Forwards and midfielders recorded much closer average loads, only differing in average distance (t(242) = 2.88, *p* = 0.0121, *g* = 0.25), but not average player load (t(242) = 1.52, *p* = 0.2849, *g* = 0.13).

Summated workloads assessed by position in the first 90 min of regulation found no significant main effect of game location, opponent rank, or game result on summated distances or player loads. A significant main effect of score spread existed for summated player load only (F(1,21) = 4.33, *p* = 0.0498), but not for distance (F(1,21) = 1.31, *p* = 0.2655).

**Figure 2:**
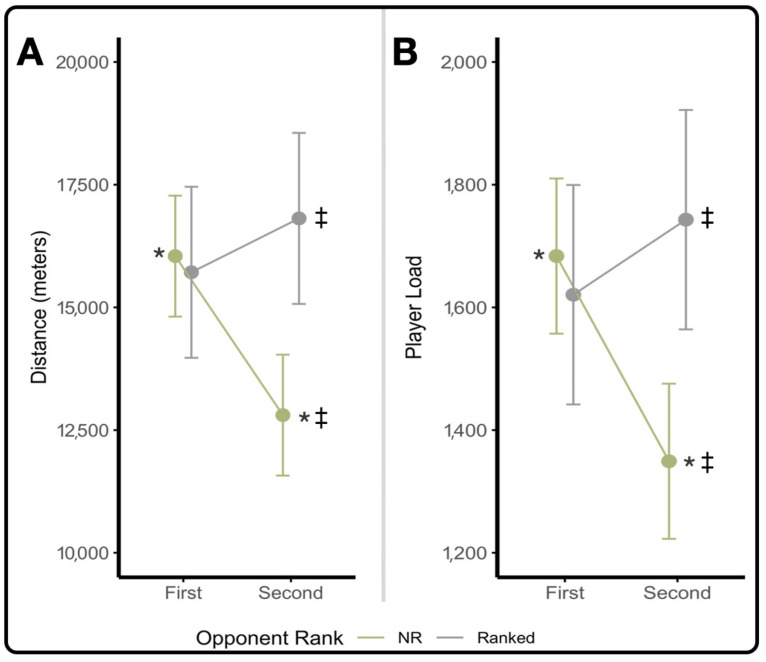
(**A**,**B**). Absolute workloads based upon opponent rank in Forwards. Least squares means plots demonstrating trends in absolute workload in forwards based upon the ranked status of the opponent. (**A**) Summated distance traveled by all forwards playing in each game. (**B**) Summated player loads recorded by all forwards playing in each game. (*) denotes significant pairwise differences between the first and second half workloads in games against non-ranked (NR) opponents, (‡) denotes significant pairwise differences in second half workloads in games against ranked opponents and non-ranked opponents.

A closer look at positional trends by half for opponent ranking and score spread demonstrate differing trends only for forwards based upon the two conditional factors. For forwards only, significant main effects of opponent rank and interactions between opponent rank*half exist on summated distance (ME of opponent rank: F(1,14) = 6.84, *p* = 0.0204, interaction opponent rank*half: F(1,14) = 9.50, *p* = 0.0081) and player loads (ME of opponent rank: F(1,14) = 5.25, *p* = 0.0380, interaction opponent rank*half: F(1,14) = 10.00, *p* = 0.0069) of all forwards during regulation, which can be seen in Figure 2A,B. These trends based upon opponent rank partially exist on average absolute workloads per player, with significant main effects of opponent rank on average distance (F(1,70) = 4.14, *p* = 0.0458), but not average player loads (F(1,70) = 3.58, *p* = 0.0625).

**Figure 3:**
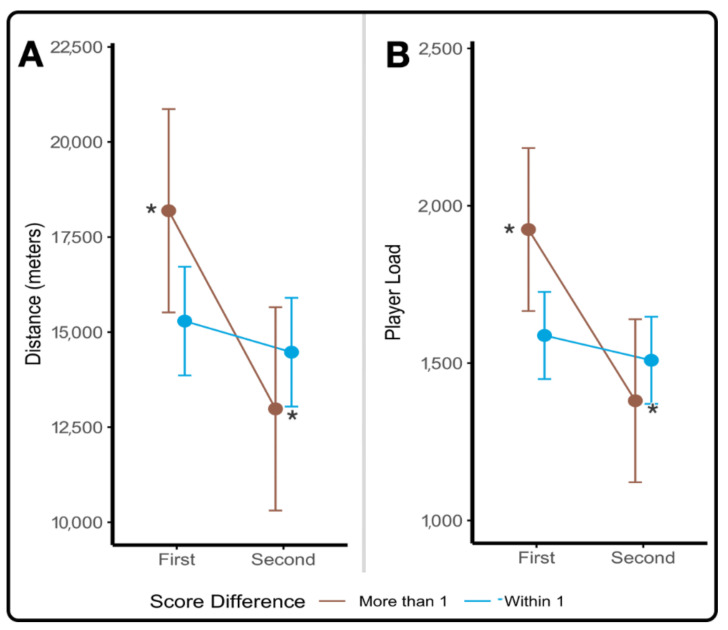
(**A**,**B**). Absolute workloads based upon score differential in Forwards. Least squares means plots demonstrating trends in absolute workload recorded in forwards based upon the final score differential. (**A**) Summated distance traveled by all forwards playing in each game. (**B**) Summated player loads recorded by all forwards playing in each game. (*) denotes significant pairwise differences between the first and second half workloads in games decided by more than one point.

Similarly, score differential was determined to impact both distance and player loads, but only in forwards. Assessment of score differential by half on summated workloads by position demonstrated significant interactions between score differential*half for cumulative distance (F(1,14) = 4.83, *p* = 0.0454) and cumulative player loads (F(1,14) = 5.77, *p* = 0.0307), which can be seen in Figure 3A,B. Similar trends exist on average absolute workloads per player, with significant main effects of score differential on average distance (F(1,70) = 5.69, *p* = 0.0198) and player loads (F(1,70) = 5.17, *p* = 0.0261).

#### 3.2.2. Relative Workload

Across all games, midfielders averaged the highest player loads per minute and meterage per minute, followed by forwards and defenders ranking the lowest. Comparatively, player load per meter was the highest in defenders and midfielders, whereas forwards measured significantly lower values. These positional averages across the season can be seen in Table 7.

**Figure 4:**
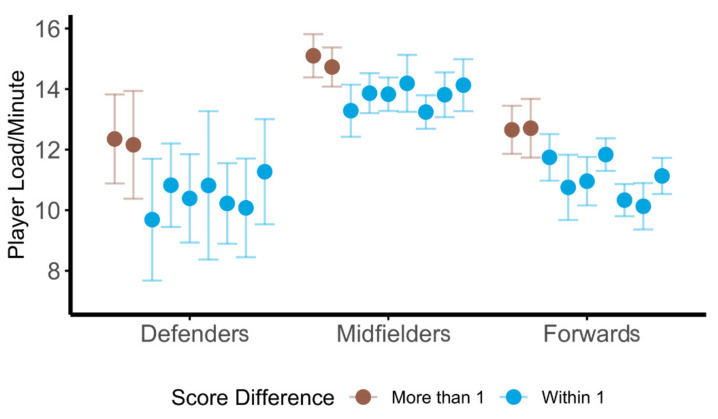
Positional player loads per minute across the season. Average player load/minute for all players during each of the nine games. Games are ordered by schedule presentation across the season.

Analysis of average relative workloads for each game were assessed for differences by position based upon game location, opponent ranking, game result, and final score differential; fixed effects for these ANOVA’s can be found in Table 8. Positional differences consistently had significant effects on all three types of relative loads, whereas game location, opponent ranking, and game result failed to significantly effect workloads. Score differential was the only other variable to significantly affect all three types of relative workloads; specifically, a pairwise comparison revealed a significant difference in M/min only for forwards between games that were decided by two or more points and games that were within one point (t(124) = 4.73, *p* < 0.0001, *g* = 0.41). Average PL/min were highest for all positions for the two games in which the final score differential was at least two points, as seen in Figure 4.

**Figure 5:**
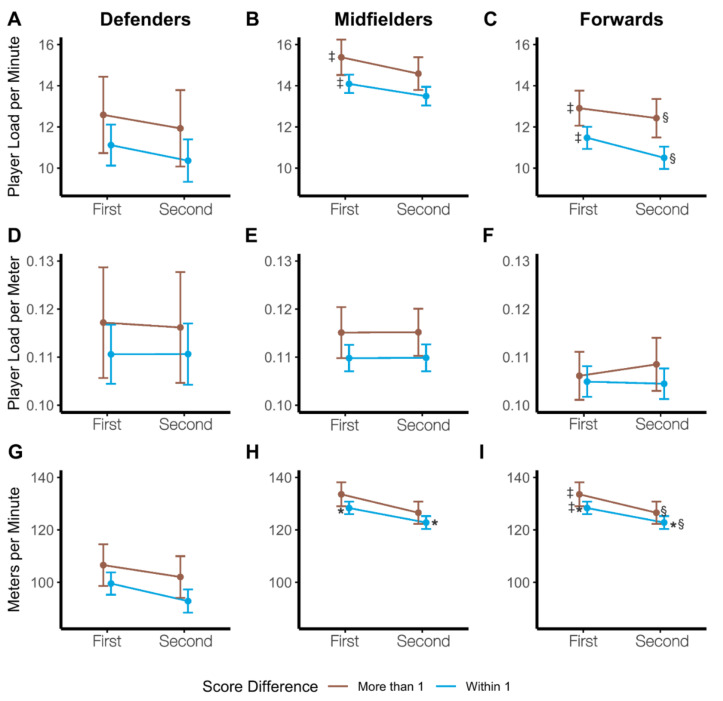
(**A**–**I**) Positional Relative Workloads by Score Differential. Least squares means plots representing trends in relative workloads by score differential across the two halves. (**A**) Player load/minute in defenders. (**B**) Player load/minute in midfielders. (**C**) Player load/minute in forwards. (**D**) Player load/meter in defenders. (**E**) Player load/meter in midfielders. (**F**) Player load/meter in forwards. (**G**) Meters/minute in defenders. (**H**) Meters/minute in midfielders. (**I**) Meters/minute in forwards. (*) denotes significant pairwise differences between the first and second halves when the game is decided by one point or less, (§) denotes significant pairwise differences in second half workloads between games won by more than one point and games decided by one point or less, (‡) denotes significant pairwise differences in first half workloads between games won by more than one point and games decided by one point or less.

A closer look into workloads based upon score differential (ANOVA’s assessing by half*score differential, individually by position) indicated that all three positions had a significant main effect of score differential for PL/min (Defenders F(1,66) = 4.12, *p* = 0.0465; Midfielders F(1,100) = 12.61, *p* = 0.0006; Forwards F(1,70) = 20.73, *p* < 0.0001) and M/min (Defenders F(1,66) = 6.44, *p* = 0.0136; Midfielders F(1,100) = 6.26, *p* = 0.0139; Forwards F(1,70) = 34.75, *p* < 0.0001); PL/M, however, was only impacted in midfielders (F(1,100) = 6.58, *p* = 0.0118), with no significant main effects existing for defenders or forwards. These trends in relative workloads can be seen in Figure 5A–I.

#### 3.2.3. Internal Workload

Across all nine games, defenders averaged a HR of 167.8 ± 9.1 BPM, midfielders averaged 171.4 ± 8.3 BPM, and forwards averaged 171.6 ± 8.1 BPM during the first 90 min of game play, with defenders having significantly lower HRs than both midfielders (t(242) = −2.76, *p* = 0.0171, *g* = 0.24) and forwards (t(242) = −2.67, *p* = 0.0218, *g* = 0.23) across the two halves.

**Figure 6:**
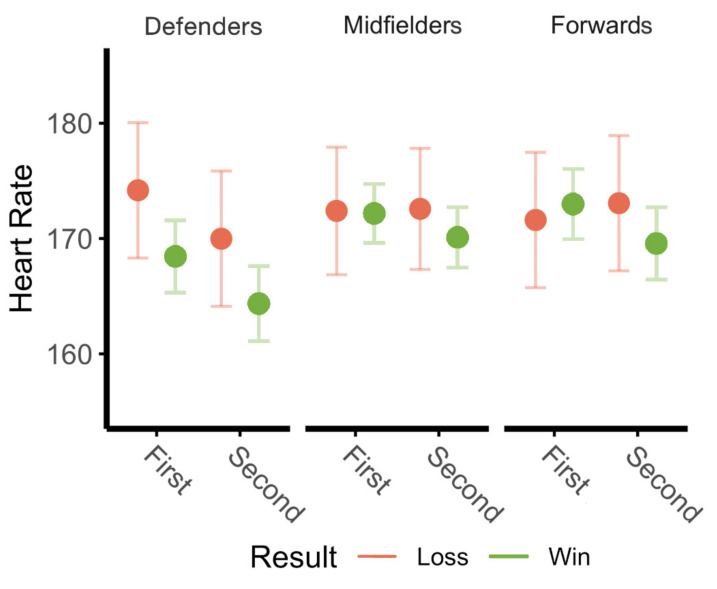
Average heart rate by game result. Average heart rates are differentiated for first and second halves based upon final game result.

Game location, opponent rank, and score differential had no effect on positional heart rates across the first and second half; however, a significant main effect of game result existed on HRs across both halves in defenders (F(1,66) = 5.22, *p* = 0.0256), but not in midfielders or forwards. Positional HRs can be seen in Figure 6 as they differ by half and game result.

## 4. Discussion

The present study examined conditional variations in team and player workloads throughout a full season of conference play of an NCAA collegiate women’s soccer team from a Power-Five Division. Having occurred during the modified “COVID-19” season, this analysis of contextual match workloads is largely unique in that it covers a competitive season involving only conference opponents, each spaced exactly one week apart. Match timing associated with the schedules of traditional seasons has been suggested to impact level of play [40]; though given the schedule evaluated herein, teams had extended and consistent periods of time to prepare for each upcoming opponent, as well as time to recover from the previous game. Notably, no differences in external or internal workloads were found to exist between home and away games. It is important to consider that COVID restrictions prevented or reduced fan attendance for all games utilized herein, which may have contributed to the absence of a “home advantage” [41,42]. Though, other contextual factors, including game result, opponent ranking, and final score differential each seemed to modify workload trends in varying ways.

Analysis of workloads reported herein was aimed toward providing perspectives surrounding contextual factors by quantifying objective workloads through numerous methods reported in the extant literature. While the contextual and positional data may be of highest value, much of the situational framework cannot be understood without the deeper analysis of workloads as they contribute to both individual players, as well as collectively in the team as a whole. Thus, average absolute values (distance and PL) suggest the average contribution per player in the game, which will ultimately be impacted by substitutions; comparatively, summated absolute values represent workloads of all contributors, independent of game durations. Relative workloads (PL/min, PL/M, M/min), which are more commonly reported in the literature, fail to demonstrate the duration of the load experienced, which can ultimately demonstrate varying degrees of vulnerability for fatigue. Workloads were assessed by game half to further support how factors such as fatigue or reduced demands may contribute to trends; similar to previous research, both external and internal workloads herein were consistently lower in the second half [9,31,32,43,44].

Existing literature on external workloads measured in competitive bouts of soccer are widely varied across levels of play, and suggest notable sex differences in performance [15]. As such, findings are inconsistent in terms of normative values, as well as the effects of various contextual factors. Even while focusing solely on the elite, and sub-elite level, variations between leagues can contribute to variations in workload, including the regulations imposed; it is important to note that there are no restrictions on numbers of substitutions in collegiate women’s soccer, and substitutions are often rather driven by depth of roster. Research looking specifically at Division I women’s soccer has reported similar relative workloads to those found herein [9,19,21,40], as well as similar positional trends in duration of play [19].

The team under review herein consistently played in a 4-3-3 formation (4 defenders, 3 midfielders, 3 forwards) during the games analyzed. Given that an extra defender is always on the field, it is relatively surprising that summated workloads of midfielders are comparable to that of defenders, though research has previously suggested midfielders to accumulate higher distances [12,14,19,44] and player loads [30,32,45,46] than defenders. These workloads can be more prominently differentiated by position in relative workloads, which account for the high degree of variation in contributions per player; similarly, midfielders demonstrated the highest player loads and distances recorded per minute. However, player loads per meter in midfielders were not significantly different than those recorded in defenders, with only forwards recording significantly lower loads. These variations in relative workloads demonstrate the variations in movement dynamics that are demanded by the different positions [47].

### 4.1. Game Result

Wins and losses appeared to be differentiated by higher absolute workloads by player, as well as altered relative workload trends across the team, which matches previously identified trends [31,32]. These trends in external workload also did not differ based upon game result for any position. It should be noted that the only two losses discussed herein were also the two games that utilized the fewest field players (three or fewer substitutions), most notably in the first half.

Interestingly, relative workloads across the team in the first half, specifically PL/min and M/min, were found to trend lower in losses as compared to wins, with the workloads in the second half relatively the same independent of result. No differences in PL/M existed based upon half; this, in combination with the higher distances and player loads during the first half of losses, suggests differing demands of the matches, with players likely sprinting further distances during losses. These workloads, however, occur independent of any psychological factors associated with the fear of losing; in both games that resulted in a loss, the first points scored by the opponent did not occur until into the second half.

While few studies have assessed heart rate during situational assessments, and rather often use subjective measures of RPE, a noteworthy trend found herein is in the heart rates of defenders. The four defenders from this team have the single-highest minutes/game on the team, playing all 90 min of regulation aside from one instance; additionally, this position group averages the lowest HRs, suggesting the degree of their endurance-trained status. Despite no significant differences in absolute or relative workloads for defenders between wins and losses, the HRs of defenders were significantly higher in both halves of games resulting in a loss compared to wins. While this was not evaluated in depth in the present study, increases in internal workload, without increases in external workload, may be suggestive of the presence of additional factors, such as anxiety [28]. Considering these players were likely experiencing the greatest attack of the season from the opponents’ offense, these games were likely accompanied by additional psychological stress. Of the two losses, one occurred in the game that determined the conference champion, which undeniably can add pressure onto all players. Considering the low sample, but important implications of this topic, situational comparisons of objective external vs. internal workloads should be further evaluated, perhaps considering heart rate variability (HRV).

### 4.2. Opponent Rank

Games against ranked opponents, which includes the two games lost in addition to a third that was won, were not found to differ from games against unranked opponents in average or summated absolute workloads, relative workloads, or HRs across the team. In contrast to the two games lost, the third game against a ranked opponent that was won took an early first half advantage (2 points) and utilized more substitutions in both halves. It is possible that this contributed to the lack of significant increase in the HR of defenders as compared to the two losses alone, as the early lead lifted some of the initial pressures for goal defense.

As noted, five substitutions were made during the first half of the successful game against ranked opponents, as compared to the three or fewer in games lost. Additionally, because the starting defenders of the team herein are rarely substituted from the bench, forwards and midfielders reap the greatest impacts from fewer substitutions. More specifically, as a position group, forwards were found to have increased distances and player loads in the second half against ranked opponents, as compared to the significant decrements that are typically seen following the first half against unranked opponents. These increased loads, likely associated with the increased pressure to score, may be suggestive of opponent rigor and match status going into the second half.

These trends in opponent rigor have been suggested by existing literature [14,31,33,34]; however, few studies have evaluated the effect of opponent rigor in such a highly ranked team. The team of evaluation in the present study was ranked (United Soccer Coaches Poll) within the top 10 of NCAA Division I teams during the fall session, and competes in a “Power 5” conference; not only does this speak for the skill of the team and their opponents, but that variations in workload still exist despite the high quality of all opponents. The perceived quality of opponents may also contribute to effort or the preparedness of athletes against ranked opponents.

### 4.3. Final Score

Analysis of final score has not been as thoroughly researched as other contextual factors discussed herein. Given the high quality of all opponents, this situational analysis demonstrated variation between close matchups and matches in which the team excelled relative to the opponent. Players across all positions averaged significantly higher relative workloads in games with higher win margins. Further, notably higher absolute workloads were found in forwards, the position with the greatest concern for scoring, to be associated with greater score spreads; both average and summated workloads recorded in the first half trended higher as compared to the first half of games that ended within one point. Of note, significant main effects of final score differential existed on nearly all the external workload variables despite the score differential at halftime being similar to most of the season’s games, which was a one-point advantage. The only games that differed in halftime score spread were the two games that resulted in a loss, which were tied at the half, and the third game against a ranked opponent, which resulted in a win (+2 at the half).

### 4.4. Implications

An eminent challenge faced by coaching staff of many sports is determining the tipping point in optimal performance between fatigued starters and rested bench players. It should be noted that the methods of external and internal workload monitoring discussed herein are not quantifiers of performance; often, games vary in required demands, which can contribute to positional loading patterns, sprinting profiles, and psychosocial impacts [48].

Theoretical aspects of training, such as, the fitness-fatigue paradigm and general adaptation syndrome, provide a helpful conceptual framework for coaches balancing various training stressors throughout a season [49,50,51]. Based on the fitness-fatigue paradigm; preparedness represents the difference between an athlete’s fitness (a generalized positive response) and fatigue (a generalized negative response), and describes an athletes’ ability to express their cumulative adaptations [49]. Preparedness has been referred to as “performance potential” and is suggestive of a higher likelihood of performing well when potential is elevated. Contrastingly, fatigue can mask an athlete’s fitness [49]; when accumulated fatigue exceeds a specific threshold, performance decrements and injury are of higher probability [52]. Poor fatigue management and non-functional overreaching can lead to mal-adaptations and unplanned, undesired diminished performance [50,51,52].

With consideration to general adaption syndrome, when a disturbance in homeostasis occurs, termed the alarm phase, various physiological mechanisms respond that lead to the resistance phase; at this time, various adaptive reserves are employed in an effort to recover, and ideally in the context of sport, recover and adapt [49,53]. Planning ahead for optimal timing in the occurrence of the alarm and resistance phases via heavy and light days allows coaches to manage fatigue in a meaningful manner during a season [53].

The balancing of fitness and fatigue in season is an inevitable challenge for coaches, particularly as it relates to playing time and substitution decisions for players with higher and lower performing capabilities. While not assessed in the present study, a contributing factor for in season maintenance considers training during the preparatory period prior to the competition phase [49,50,51]; it is important to note that due to COVID-19, preseason training during the season herein deviated in duration and structure from that of a normal season. Properly navigating a season however, is not as easy as simply avoiding too much fatigue; athletes must also be fit to perform at a high level, specifically possessing metabolic, strength-power and speed abilities. Thus, training, practicing, and competing in a manner in which these abilities do not diminish longitudinally is vital for maintaining stress tolerance throughout the season [49].

### 4.5. Limitations and Future Research

The present study was conducted during a shortened training and competitive season, among many other abnormalities as compared to a normal season. As mentioned previously, a majority of games were completed in the absence of fans, which may have diminished the “home advantage” that has been found elsewhere [41,42,54,55]. Aside from the increased consistency and time between scheduled matches, numerous modifications during the COVID-19 sports season imposed additional challenges on team success [56]. For one, the amount of pre-season training that teams were allowed to participate in was greatly reduced from traditional fall season preparation. Second, for the first time ever, active collegiate players were watching their teammates play live, including games in their home stadium, from a TV screen; arguably the largest challenge of this season was the constant rotation of unexpected ineligibility, which would be re-granted in 14 days or less. As such, the plasticity of starting lineups required a degree of flexibility that coaches and players have never experienced. Contextual factors characteristic of this novel season, in addition to the high degree of variability in individual player contributions, may have conduced trends found herein.

Further evaluation of these variables throughout a traditional season is of value for increasing the application of findings, as well as increasing monitoring opportunities; while 130 records were recorded across all nine games, a typical season has three times the number of matches studied herein, providing greater depth of the data relative to the varying context. The relatively low sample size of 18 players is unlikely to increase in a traditional season, which is a widely occurring limitation of GPS workload studies [9,11,12,14,21,22,40,57].

Rather than attempting to assess workloads across various teams or successive years to increase sample size, both of which are likely to increase variability in the data, a deeper dissection of team-wide data connecting workload to performance would be of greater benefit [26]. A more holistic approach should be taken that encompasses the contextual relationships between external and internal workloads with respect to acute preparedness and performance fatigue [29]. Additional considerations for HRV, opportunity recognition, active skill success, or the dynamics of communication with other players, among many others, would be valuable for future research. Such analyses are limited, and are likely to require considerable resources to execute, but provide much greater insight on context-based performance.

## 5. Conclusions

Of the many important in-game decisions required of coaches, choosing what athletes should be on the field at various points throughout a game can be one of the most critical. Coaches are often pressured to play [what they deem to be] their best lineup, which is aimed toward optimizing acute performance, while also considering fatigue and performance ramifications across a season. Closer games likely facilitate greater levels of stress as compared to games spent enjoying a comfortable lead. For this reason, a team’s depth is incredibly impactful in that a “deeper bench” minimizes the assumed drop off in performance between starters and non-starters, likely reducing a coaches’ concern for resting starters at various points during a game and throughout a season.

In the present study, numerous trends in workloads were explored for their impact on in-game movement dynamics, which builds upon existing literature that has aimed to focus on a single variable rather than how multiple facets may co-exist across a season. It was found that trends in workload varied based upon the context of the game, and specifically the positional groups that tend to be the most impacted by that game state. Additionally, the two games that resulted in losses involved the fewest player substitutions. The observational nature of the data is important to appreciate in that cause-and-effect relationships cannot be established; however, this type of real-world data, when contextualized, can add to the body of existing literature and enhance knowledge on the role that context plays in human performance outcomes. As contextual variations such as these are explored over time and across similar sample groups, it is hoped that it can aid coaches in their ability to make informed decisions. Garnering a better understanding of game demands and resultant internal stress responses may aid the coach in encouraging additional considerations for sensitive decisions regarding their lineup and game plans with respect to both short and long-term success.

## Figures and Tables

**Table 1:** Game Descriptive Information and Results.

Schedule	Opponent Rank	Game Location	Final	Result	Point Spread	Players in 1st Half	Players in 2nd Half	Players Total
Game 1	NR	Away	Reg	Win	2	16	15	17
Game 2	NR	Home	Reg	Win	3	14	15	15
Game 3	6	Away	2OT	Loss	−1	13	13	13
Game 4	NR	Home	Reg	Win	1	14	14	14
Game 5	NR	Away	Reg	Win	1	14	13	14
Game 6	NR	Home	Reg	Win	1	14	14	15
Game 7	NR	Away	Reg	Win	1	14	13	14
Game 8	11	Home	Reg	Win	1	15	12	15
Game 9	3	Away	Reg	Loss	−1	12	13	13

Information and final results for each game included in analysis. [NR, not ranked; Reg, regulation; 2OT, second overtime].

**Table 2:** Fixed Effects on Absolute Workloads.

Context		Distance		Player Load	
	Fixed Effect	Average	Summated	Average	Summated
Home vs. Away	ME of Game Location	F(1,117.8) = 0.01, *p* = 0.9273	F(1,14) = 0.57, *p* = 0.4622	F(1,121.7) = 0.11, *p* = 0.7452	F(1,14) = 0.04, *p* = 0.8517
	ME of Half	F(1,111.5) = 2.19, *p* = 0.1421	F(1,14) = 4.93, *p* = **0.0434**	F(1,114.2) = 2.54, *p* = 0.1135	F(1,14) = 2.41, *p* = 0.1431
	Game Location*Half	F(1,111.5) = 0.02, *p* = 0.8857	F(1,14) = 0.34, *p* = 0.5674	F(1,114.2) = 0.02, *p* = 0.8957	F(1,14) = 0.18, *p* = 0.6765
Ranked vs. Unranked Opponents	ME of Opponent Rank	F(1,117.7) = 3.54, *p* = 0.0622	F(1,14) = 0.49, *p* = 0.4978	F(1,121.8) = 1.98, *p* = 0.1616	F(1,14) = 0.01, *p* = 0.9199
	ME of Half	F(1,111.4) = 0.72, *p* = 0.3980	F(1,14) = 3.00, *p* = 0.1053	F(1,114.2) = 0.82, *p* = 0.3660	F(1,14) = 1.31, *p* = 0.2721
	Opponent Rank*Half	F(1,111.4) = 1.91, *p* = 0.1696	F(1,14) = 1.20, *p* = 0.2921	F(1,114.2) = 2.41, *p* = 0.1232	F(1,14) = 0.92, *p* = 0.3551
Wins vs. Losses	ME of Game Result	F(1,116) = 4.75, *p* = **0.0313**	F(1,14) = 1.12, *p* = 0.3082	F(1,120.3) = 4.68, *p* = **0.0326**	F(1,14) = 1.15, *p* = 0.3009
	ME of Half	F(1,109.7) = 0.63, *p* = 0.4279	F(1,14) = 0.83, *p* = 0.3773	F(1,112.8) = 0.58, *p* = 0.4466	F(1,14) = 0.27, *p* = 0.6124
	Game Result*Half	F(1,109.7) = 0.54, *p* = 0.4638	F(1,14) = 4.22, *p* = 0.0590	F(1,112.8) = 0.93, *p* = 0.3374	F(1,14) = 2.54, *p* = 0.1331
Score Differential	ME of Score Differential	F(1,118.8) = 1.13, *p* = 0.2893	F(1,14) = 1.81, *p* = 0.2002	F(1,122.5) = 0.15, *p* = 0.6952	F(1,14) = 4.69, *p* = **0.0481**
	ME of Half	F(1,112.5) = 3.52, *p* = 0.0632	F(1,14) = 4.98, *p* = **0.0424**	F(1,115.1) = 4.10, *p* = **0.0452**	F(1,14) = 3.06, *p* = 0.1022
	Score Differential*Half	F(1,112.5) = 1.40, *p* = 0.2401	F(1,14) = 0.40, *p* = 0.5360	F(1,115.1) = 1.57, *p* = 0.2122	F(1,14) = 0.28, *p* = 0.5036

ANOVA table for the fixed effects for contextual variable analyses of absolute workload variables, which each include two main effects and an interaction. Significant fixed effects are in bold. [ME, main effect]. * demonstrates the interaction between the two variables

**Table 3:** Average Relative Workload Variables.

Context	Condition	Player Load per Minute	Player Load per Meter	Meterage per Minute
*All Games*		12.447 ± 2.38	0.1096 ± 0.012	113.15 ± 15.85
*Game Location*	Home	12.497 ± 2.51	0.1088 ± 0.012	114.43 ± 17.11
	Away	12.406 ± 2.28	0.1103 ± 0.011	112.10 ± 14.72
*Opponent Rank*	Ranked	12.106 ± 2.32	0.1088 ± 0.012	110.83 ± 14.61
	Unranked	12.603 ± 2.40	0.1100 ± 0.011	114.21 ± 16.33
*Result*	Wins	12.489 ± 2.44	0.1093 ± 0.011	113.83 ± 16.56
	Losses	12.285 ± 2.17	0.1109 ± 0.013	110.50 ± 12.54
*Score Differential*	Within 1	12.117 ± 2.35	0.1086 ± 0.012	111.24 ± 16.07
	Greater than 1	13.480 ± 2.22	0.1129 ± 0.011	119.11 ± 13.63

Values are presented as Mean ± SD and are averaged across players in the first 90 min of each game.

**Table 4:** Fixed Effects on Relative Workloads.

Context	Fixed Effect	Player Load per Minute	Player Load per Meter	Meterage per Minute
Home vs. Away	ME of Game Location	F(1,127.4) = 0.00, *p* = 0.9931	F(1,128.3) = 0.73, *p* = 0.3950	F(1,126.2) = 0.42, *p* = 0.5169
	ME of Half	F(1,116.8) = 52.92, ***p* < 0.0001**	F(1,116.9) = 0.69, *p* = 0.4090	F(1,116.3) = 66.67, ***p* < 0.0001**
	Game Location*Half	F(1,116.8) = 2.07, *p* = 0.1530	F(1,116.9) = 0.29, *p* = 0.5920	F(1,116.3) = 2.40, *p* = 0.1244
Ranked vs. Unranked Opponents	ME of Opponent Rank	F(1,127.4) = 1.02, *p* = 0.3147	F(1,128.3) = 0.26, *p* = 0.6105	F(1,126.2) = 1.08, *p* = 0.3004
	ME of Half	F(1,116.8) = 41.33, ***p* < 0.0001**	F(1,116.9) = 0.25, *p* = 0.6153	F(1,116.3) = 53.68, ***p* < 0.0001**
	Opponent Rank*Half	F(1,116.8) = 0.19, *p* = 0.6661	F(1,116.9) = 0.35, *p* = 0.5580	F(1,116.3) = 0.02, *p* = 0.8760
Wins vs. Losses	ME of Game Result	F(1,126.9) = 0.07, *p* = 0.7872	F(1,128) = 0.60, *p* = 0.4404	F(1,125.7) = 0.87, *p* = 0.3528
	ME of Half	F(1,116.2) = 16.53, ***p* < 0.0001**	F(1,116.6) = 0.02, *p* = 0.8810	F(1,115.5) = 22.47, ***p* < 0.0001**
	Game Result*Half	F(1,116.2) = 12.32, ***p* = 0.0006**	F(1,116.6) = 1.90, *p* = 0.1713	F(1,115.5) = 13.22, ***p* = 0.0004**
Score Differential	ME of Score Differential	F(1,127.6) = 8.67, ***p* = 0.0038**	F(1,128.4) = 3.14, *p* = 0.0786	F(1,126.7) = 7.13, ***p* = 0.0086**
	ME of Half	F(1,117.2) = 32.59, ***p* < 0.0001**	F(1,117) = 0.02, *p* = 0.8805	F(1,117) = 42.69, ***p* < 0.0001**
	Score Differential*Half	F(1,117.2) = 0.44, *p* = 0.5094	F(1,117) = 0.97, *p* = 0.3272	F(1,117) = 0.24, *p* = 0.6227

ANOVA table for the fixed effects for contextual variable analyses of relative workload variables, which each include two main effects and an interaction. Significant fixed effects are in bold. [ME, main effect]. * demonstrates the interaction between the two variables

**Table 5:** Fixed Effects on Average Heart Rate.

Context	Fixed Effect	Average Heart Rate
Home vs. Away	ME of Game Location	F(1,126) = 0.07, *p* = 0.7855
	ME of Half	F(1,116.1) = 20.13, ***p* < 0.0001**
	Game Location*Half	F(1,116.1) = 0.85, *p* = 0.3580
Ranked vs. Unranked Opponents	ME of Opponent Rank	F(1,125.9) = 0.36, *p* = 0.5521
	ME of Half	F(1,116.1) = 15.10, ***p* = 0.0002**
	Opponent Rank*Half	F(1,116.1) = 0.37, *p* = 0.5443
Wins vs. Losses	ME of Game Result	F(1,124.6) = 1.81, *p* = 0.1808
	ME of Half	F(1,114.7) = 7.58, ***p* = 0.0069**
	Game Result*Half	F(1,114.7) = 2.50, *p* = 0.1167
Score Differential	ME of Score Differential	F(1,126.4) = 0.03, *p* = 0.8664
	ME of Half	F(1,116.6) = 17.38, ***p* < 0.0001**
	Score Differential*Half	F(1,116.6) = 0.57, *p* = 0.4502

ANOVA table for the fixed effects for contextual analyses of internal workload quantified via average heart rate, which each include two main effects and an interaction. Significant fixed effects are in bold. [ME, main effect]. * demonstrates the interaction between the two variables.

**Table 6:** Summated Workload Metrics by Position.

Workload	Game Half	Defenders	Midfielders	Forwards
Total Distance (m)	First	18,967 ± 1121	17,537 ± 1234	15,935 ± 1643
	Second	16,339 ± 2695	18,837 ± 1892	14,141 ± 2193
Total Player Load (au)	First	2148 ± 168	1942 ± 123	1663 ± 180
	Second	1846 ± 357	2101 ± 236	1480 ± 209

Values presented as Mean ± SD represent cumulative sums from all players of that position during the half. [au, arbitrary units; m, meters].

**Table 7:** Average Relative Workloads by position.

Position	Player Load/Minute	Player Load/Meter	Meters/Minute
Defenders	10.95 ± 2.6 ^b^	0.112 ± 0.016 ^c^	97.25 ± 11.4 ^b,c^
Midfielders	14.06 ± 1.4 ^a,c^	0.111 ± 0.009 ^c^	126.42 ± 7.1 ^a,c^
Forwards	11.45 ± 1.4 ^b^	0.105 ± 0.008 ^a,b^	108.60 ± 8.6 ^a,b^

Values presented as Mean ± SD (^a^ significantly different from defenders, *p* < 0.05; ^b^ significantly different from midfielders, *p* < 0.05; ^c^ significantly different from forwards, *p* < 0.05).

**Table 8:** Fixed Effects on Positional Relative Workloads.

Context	Fixed Effect	Player Load/Minute	Player Load/Meter	Meters/Minute
Home vs. Away	ME of Game Location	F(1,124) = 0.00, *p* = 0.9557	F(1,124) = 0.66, *p* = 0.4170	F(1,124) = 0.78, *p* = 0.3804
	ME of Position	F(2,124) = 37.94, ***p* < 0.0001**	F(2,124) = 3.81, ***p* = 0.0248**	F(2,124) = 124.11, ***p* < 0.0001**
	Game Location*Position	F(2,124) = 0.07, *p* = 0.9372	F(2,124) = 0.03, *p* = 0.9725	F(2,124) = 0.54, *p* = 0.5859
Ranked vs. Unranked Opponents	ME of Opponent Rank	F(1,124) = 1.95, *p* = 0.1653	F(1,124) = 0.42, *p* = 0.5208	F(1,124) = 3.60, *p* = 0.0602
	ME of Position	F(2,124) = 33.92, ***p* < 0.0001**	F(2,124) = 3.49, ***p* = 0.0335**	F(2,124) = 107.65, ***p* < 0.0001**
	Opponent Rank*Position	F(2,124) = 0.17, *p* = 0.8481	F(2,124) = 0.16, *p* = 0.8563	F(2,124) = 0.62, *p* = 0.5403
Wins vs. Losses	ME of Game Result	F(1,124) = 0.06, *p* = 0.8117	F(1,124) = 0.68, *p* = 0.4125	F(1,124) = 2.21, *p* = 0.1394
	ME of Position	F(2,124) = 21.94, ***p* < 0.0001**	F(2,124) = 2.55, *p* = 0.0824	F(2,124) = 73.07, ***p* < 0.0001**
	Game Result*Position	F(2,124) = 0.10, *p* = 0.9013	F(2,124) = 0.01, *p* = 0.9941	F(2,124) = 0.27, *p* = 0.7669
Score Differential	ME of Score Differential	F(1,124) = 16.65, ***p* < 0.0001**	F(1,124) = 4.06, ***p* = 0.0461**	F(1,124) = 27.87, ***p* < 0.0001**
	ME of Position	F(2,124) = 28.44, ***p* < 0.0001**	F(2,124) = 4.38, ***p* = 0.0145**	F(2,124) = 96.19, ***p* < 0.0001**
	Score Differential*Position	F(2,124) = 0.35, *p* = 0.7041	F(2,124) = 0.29, *p* = 0.7473	F(2,124) = 3.27, ***p* = 0.0414**

ANOVA table for the fixed effects for analyses exploring contextual variable and positional effects on relative workload variables, which each include two main effects and an interaction. Significant fixed effects are in bold. [ME, main effect]. * demonstrates the interaction between the two variables.

## Data Availability

Data cannot be shared publicly because certain elements of the data are owned by a third party and are considered confidential. Data are available from the RNI Institutional Data Access/Ethics Committee (contact via Padmashree Tirumalai, padma.tirumalai@hsc.wvu.edu) for researchers who meet the criteria for access to confidential data.
